# Breech birth at home: outcomes of 60 breech and 109 cephalic planned home and birth center births

**DOI:** 10.1186/s12884-018-2033-5

**Published:** 2018-10-11

**Authors:** Stuart James Fischbein, Rixa Freeze

**Affiliations:** 1 Birthing Instincts, Inc., Los Angeles, CA USA; 2 Wabash College, 211 Center Hall, Crawfordsville, IN 47933 USA

**Keywords:** Breech, Vaginal breech delivery, Delivery mode, Home birth, Birth center, Out-of-hospital birth, Upright birth, Physiological birth, Autonomy, Informed consent

## Abstract

**Background:**

Research on outcomes of out-of-hospital breech birth is scarce. This study evaluates the outcomes of singleton term breech and cephalic births in a home or birth center setting.

**Methods:**

This is a retrospective observational cohort study of 60 breech and 109 cephalic planned out-of-hospital term singleton births during a 6 year period with a single obstetrician. Outcomes measured included mode of delivery; birth weights; 1 & 5-min Apgar scores; ante-, intra-, and post-partum transports; perineal integrity; and other maternal and neonatal morbidity.

**Results:**

50 breech and 102 cephalic presentations were still in the obstetrician’s care at the onset of labor; of those, 10 breech and 11 cephalic mothers required transport during labor. 76% of breech and 92.2% of cephalic births were planned to occur at home, with the remainder at a freestanding birth center. When compared to the cephalic group, the breech group had a higher rate of antepartum and in-labor transfer of care and cesarean section. Among completed out-of-hospital births, the breech group had a significantly higher rate of 1-min Apgar scores < 7 but no significant difference at 5 min. Rates of vaginal birth for both groups were high, with 84% of breech and 97.1% of cephalic mothers giving birth vaginally in this series. Compared to primiparas, multiparas in both groups had less perineal trauma and higher rates of out-of-hospital birth, vaginal birth, and spontaneous vaginal birth. No breech infant or mother required postpartum hospital transport, while one cephalic infant and one cephalic mother required postpartum transport. Of the babies born out-of-hospital, there was one short-term and one longer-term birth injury among the breech group and one short-term brachial plexus injury in the cephalic group.

**Conclusions:**

A home or birth center setting leads to high rates of vaginal birth and good maternal outcomes for both breech and cephalic term singleton presentations. Out-of-hospital vaginal breech birth under specific protocol guidelines and with a skilled provider may be a reasonable choice for women wishing to avoid a cesarean section—especially when there is no option of a hospital breech birth. However, this study is underpowered to calculate uncommon adverse neonatal outcomes.

## Background

The options for term vaginal breech birth (VBB) have rapidly declined in the western world. This decline began in the 1980s and has led to the removal of training of breech skills from most residency programs. This trend was codified, in large part, by the 2000 Term Breech Trial (TBT), which found poorer outcomes for planned vaginal breech births compared to planned cesareans for term singleton babies [1]. Counseling for term breech pregnancies often steers women towards cesarean section and only addresses short-term risks to the baby [2]. The TBT has drawn criticism for flaws in its study design, case selection, and intrapartum care practices across the 174 participating centers [3–6]. In addition, the 2-year follow-up study found no long-term difference in death or neurodevelopmental delay among a subset of the overall TBT cohort [7].

Since that time there have been well over 100 single-center, multi-center, and birth certificate/national registry studies published on term breech outcomes, some of which recommend routine cesarean and others which support the option of a planned vaginal birth. The RCOG’s 2017 breech guideline is the most up-to-date compilation of the body of post-TBT literature [8]. The most influential study since the TBT has been the 2006 PREMODA study, a multicenter prospective observational study of 2526 planned vaginal and 5579 planned cesareans births [9]. With a cohort nearly four times the size of the TBT and with strict selection criteria and protocols, the PREMODA study did not find any significant differences in outcomes between planned vaginal breech birth (pVBB) and planned cesarean section (pCS).

Cochrane reviews have also moved from certainty regarding recommended mode of delivery for term breech immediately following the TBT [10] to complex uncertainty, acknowledging in 2015 that performing a cesarean should “be weighed against factors such as the mother’s preferences for vaginal birth and risks such as future pregnancy complications in the woman’s specific healthcare setting” [11]. The 2015 review also noted that the TBT data are not generalizable to dissimilar settings or in places where delivery techniques and protocols “differ materially.” Due to these developments since the TBT, the national obstetric societies of the USA (ACOG), Canada (SOGC), UK (RCOG), and Australia & New Zealand (RANZCOG) have all reversed their early-2000 guidelines recommending routine cesarean beginning in 2006 and now currently support properly selected VBB for term singleton fetuses [8, 12–14].

The 2015 Cochrane review also recommended research on how to “improve the safety of breech delivery.” The most notable research and innovations have come from midwives and obstetricians around the world exploring breech birth with mothers in upright positions [15–24]. In particular, Louwen’s 2017 study of 740 term breech births in Frankfurt (433 pVBB and 314 pCS) found that upright vaginal breech birth leads to a shorter 2nd stage, fewer cesareans, less intervention, fewer maneuvers, and fewer injuries to mother and baby, compared to on-the-back positions [15]. In addition to research indicating that cesarean section might not be the universal solution for term breech presentation, there is growing awareness of the importance of vaginal birth and of the risks of cesarean section on the long-term health of the baby and mother [25–30].

Medical ethics recognizes that patient autonomy in decision making should be honored [31]. In 2016 ACOG produced a committee opinion strongly supporting maternal autonomy, including the right of pregnant women to refuse a recommended treatment [32]. Despite good evidence, ethical arguments, and organizational support for the option of vaginal breech birth, there has been a concerted effort to eliminate VBB in most American hospitals, including outright bans. As hospitals continue to restrict or ban vaginal breech birth, some women will give birth at home or in birth centers to avoid a mandatory cesarean section. Some women also choose to give birth unassisted (with no care provider). Some state legislatures, including in CA where SJF practices, have recently restricted midwives from attending OOH breech births, further narrowing women’s options.

Research on outcomes of planned breech home or birth center birth is sparse; the two main datasets that include a subset of home breech births are the MANA Statistics Project and the National Vital Statistics System Natality Data Files [33–36]. Both show an increased risk of adverse outcomes for breech birth at home compared to cephalic babies. Citing the outcomes reported in Cheyney 2014 [36], ACOG considers fetal malpresentation an absolute contraindication to planned home birth [37]. These datasets did not have information about practitioner education or skill level in breech, selection criteria, labor management protocols, or maternal motivations for seeking an out-of-hospital breech birth. Without this information, it is difficult to determine what causes the higher rates of adverse outcomes. Our study examines outcomes for vaginal breech birth outside of a hospital for well-selected women attended by an experienced practitioner.

## Methods

This paper is a retrospective analysis of a series of planned out-of-hospital births: 60 term breech and 109 term cephalic presentations. All were under the care of a single obstetrician and occurred between August 2010 and April 2017. We excluded VBACs from this analysis to eliminate the confounding factor of a scarred uterus; we hope to analyze both cephalic & breech VBACs in a separate paper. The birth team consisted of an obstetrician (SJF), a licensed midwife, and a midwifery student. SJF has been in private practice in greater Los Angeles since 1986 and has attended close to 200 vaginal breech births. In 2010, SJF’s admitting hospital instituted a breech and VBAC ban, prompting SJF to continue offering these birth options in an out-of-hospital setting.

Equipment brought to each birth included IV fluids and tubing, sterile gloves, gauze, pads, betadine, suture material, and instruments. The birth team also supplied an inflatable birth pool. Medications included antibiotics, lidocaine, oxytocin, misoprostol, oral methylergonovine, vitamin K, and oxygen. In this series SJF also carried a portable GE Voluson ultrasound, a Masimo pulse oximeter, a Mityvac vacuum, Piper forceps, Simpson forceps, and Tucker-McLean forceps. All licensed practitioners were certified in neonatal resuscitation and cardio-pulmonary resuscitation.

The women in this series were all in good health and received prenatal care with an obstetrician, a midwife, or a collaboration of both. Most of the cephalic clients self-selected the option of home birth at an early gestational age and experienced continuity of care throughout their pregnancies. In contrast, most of the breech mothers entered into SJF’s care late in pregnancy after discovering the breech presentation and after unsuccessful attempts to turn the baby.

Most women with breech babies tried chiropractic Webster technique, acupuncture with moxabustion, inversions, and Spinning Babies exercises [38]. Most were offered external cephalic version (ECV). Clients who were not good candidates, who declined ECV, or for whom ECV was unsuccessful were counseled regarding all available options for giving birth to their breech baby. In the Los Angeles metropolitan area, this included scheduled cesarean section (easily available), cesarean section at the onset of labor (not easily available), vaginal breech birth in a hospital where breech-skilled physicians were on call intermittently (rarely available), or an OOH breech birth with SJF (available 24/7 except when he was out of town).

Women in both cohorts were not excluded for conditions that were unlikely to affect labor such as diet-controlled gestational diabetes, mild chronic hypertension, or age over 35. Cephalic babies did not have an upper estimated fetal weight (EFW) limit, while breech babies did. Planned OOH births could occur from 36 weeks + 0 days onward. The data were not analyzed prior to completion of the 50th breech birth.

Term breech clients were selected and labor was managed according to these 8 basic criteria (asterisks indicate criteria shared among cephalic clients):

1. Frank or complete breech presentation
2. Flexed or neutral head (confirmed by ultrasound)
3. EFW between 5 and 9.5lbs (~ 2250–4300 g)
4. Clinically adequate maternal pelvis by history and/or exam
5. No gross anomalies*
6. Spontaneous labor; no induction or augmentation*
7. Fetal and maternal tolerance of labor*
8. Well-informed and motivated parents*

The midwifery model of care encourages settings where women feel “free, safe, and private” [39]. Breech and cephalic labors were managed identically with two minor exceptions: for breech, water birth was discouraged and an initial vaginal exam was offered upon SJF’s arrival. In all 152 labors women were encouraged to eat and drink, ambulate, change positions, and choose their birth location and position. Both breech and cephalic women had the option of a shower and tub for labor analgesia; water birth for breech births was not preferred due the higher likelihood of assistance.

Fetal monitoring was performed intermittently with a Nicolet Elite 200 Handheld Doppler. Auscultation was individualized but the usual protocol was every 30–60 min in active labor, every 15–30 min in transition and every 5–10 min in second stage. With the breech labors SJF offered an initial vaginal exam in labor to confirm fetal position. Otherwise, vaginal exams were kept to a minimum, often withheld until maternal guttural vocalizations signaled an urge to push.

Pushing only began when maternal urge became irresistible; pushing was spontaneous rather than coached. Breech mothers were encouraged to labor down before active pushing began. Passage of pasty meconium was considered a positive sign of descent. Breech mothers were counseled about the benefits of upright and hands-off techniques. On-the-back positioning for breech was used on an as-needed basis and with maternal consent. Delayed cord clamping (usually until the placenta was birthed or later) and immediate and uninterrupted skin-to-skin were routine for both groups. Footage of a primip frank breech birth with SJF is available to view at https://vimeo.com/45678615 [40]. The baby was 39 ½ weeks gestation, weighed 2890 g, and had 1 & 5 min Apgars of 8 and 9.

This project received approval from the Wabash College IRB (IRB# 1610303). Written or verbal consent from participants was not required since the project used de-identified data extracted from medical records.

Data were analyzed using Stata version 14.1. We employed Fisher’s exact test for categorical data and t-test for continuous data. A *p*-value of < 0.05 was considered statistically significant.

## Results

**Fig. 1:**
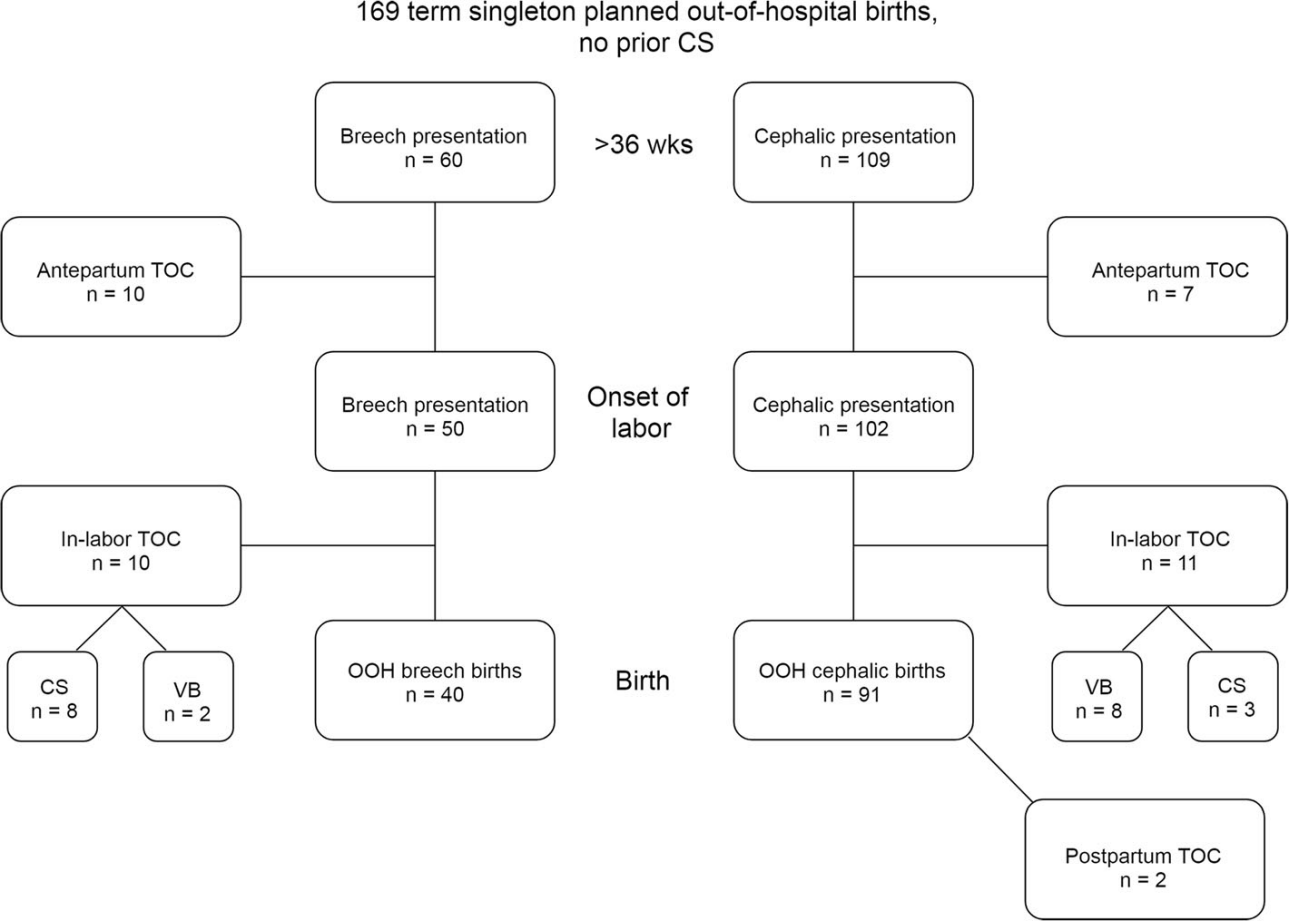
Flow of OOH clients from > 36 weeks to postpartum. This figure shows the flow of SJF’s clients from > 36 weeks to postpartum, including pre-labor, in-labor, and postpartum transfers of care

A total of 169 pregnant women entered into care at term (see Fig. 1). Antepartum transfer of care (TOC) occurred for 10 breech mothers (16.7%) and 7 cephalic mothers (6.4%; see Table 1). The cephalic mother complaining of decreased fetal movement at 37 weeks had a poor biophysical profile and underwent an urgent cesarean section. The IUFD at 39 weeks was due to an avulsion of a velamentous umbilical cord insertion.

**Table 1:** Reasons for antenatal transfer of care to a hospital-based provider

Indication for transfer	Breech *n* = 60	Cephalic *n* = 109
> 41 wks, suspected macrosomia	1	
Cholestasis	1	
SJF out of town	3	
Oligohydramnios	2	
SPROM > 5 days, NIL (not in labor)	1	
> 42 wks, NIL	2	1
37 wks, preeclampsia		1
39 wks, IUFD		1
43 wks, polyhydramnios		1
39 wks, increasing hypertension		1
37 wks, decreased fetal activity, NIL		1
DVT (blood clot)		1
Total antenatal transfers	10 (16.7%)	7 (6.4%)

After these 17 transfers of care, there were 50 breech and 102 cephalic full-term women still under SJF’s care when they went into spontaneous labor. Table 2 presents maternal, fetal, and obstetric characteristics of these two groups at the onset of labor.

**Table 2:** Maternal, fetal, and obstetric characteristics of the study population at onset of labor

Characteristic	Breech *n* = 50	Cephalic *n* = 102	*P* value
Parity			0.006
Primipara	41 (82.0%)	59 (57.8%)	
Multipara	9 (18.0%)	43 (42.2%)	
Type of breech			
Frank	45 (90.0%)	NA	
Complete	5 (10.0%)	NA	
Planned location of birth			
Home	38 (76.0%)	94 (92.2%)	
Birth center	12 (24.0%)	8 (7.9%)	
Mean birthweight (range)^a^	3274 g (2410–4224)	3606 g (2325–5046)	0.0002
Birthweight percentiles^b^			
< 10th	10 (20.8%)	11 (11.0%)	
> 90th	1 (2.1%)	16 (16.0%)	
Mean wks gestation (range)^c^	39.9 (37–42)	40.1 (35–44)	0.154

^a^ Birthweights were not recorded for 2 cephalic and 2 breech infants; one infant from each group was a hospital transport

^b^ Compared against percentile tables in Talge 2014 [69]

^c^ Weeks gestation were recorded as a rounded average, i.e. 37 weeks = 36 4/7 to 37 3/7

A significantly higher proportion of the breech group were first-time mothers compared to the cephalic group. The mean gestational age was not statistically significant. Breech babies had a significantly lower mean birthweight than cephalic babies (3274 g vs 3606 g), possibly due to the upper EFW limit for breech but not for cephalic presentations. This trend is visible in the number of babies below the 10th or above the 90th birth weight percentiles.

57% of the breech babies were female, similar to a reported rate of 56.7% among Dutch babies born during the 40th gestational week [41]. We are missing complete data on maternal age, pre-pregnancy weight, pregnancy weight gain, and length of 1st stage and therefore did not include those factors in this analysis.

Table 3 examines the location of birth and the mode of birth for both groups, sorted by parity. The overall cesarean rate was 16% in the breech group and 2.9% in the cephalic group; all cesareans were among primiparous clients. All multips in both groups had 100% rates of spontaneous vaginal birth. Of the 41 breech primips, 31 (75.6%) gave birth vaginally OOH, compared to 50 of the 60 cephalic primips (83.3%). Four breech primips were assisted with Piper forceps, all in the early years of the series before SJF adopted upright breech techniques. Upright positioning for breech birth (hands & knees, kneeling, or standing) occurred in 11 (27.5%) of the forty successful OOH breech births, while the other 29 (72.5%) used a modified lithotomy position.

**Table 3:** Location of birth and mode of birth for breech & cephalic mothers, categorized by parity

	Breech primip *n* = 41	Breech multip *n* = 9	Total breech *n* = 50	Cephalic primip *n* = 60	Cephalic multip *n* = 42	Total cephalic *n* = 102
Birth location						
OOH	31 (75.6%)	9 (100%)	40 (80.0%)	50 (83.3%)	41 (97.6%)	91 (89.2%)
Hospital (TOC)	10 (24.4%)	0	10 (20.0%)	10 (16.7%)	1 (2.4%)	11 (10.8%)
Mode of birth						
Cesarean	8 (19.5%)	0	8 (16.0%)	3 (5.0%)	0	3 (2.9%)
Vaginal	33 (80.5%)	9 (100%)	42 (84.0%)	57 (95.0%)	42 (100%)	99 (97.1%)
*SVD*	*27 (65.9%)*	*9 (100%)*	*36 (72.0%)*	*37 (61.7%)*	*42 (100%)*	*79 (77.5%)*
*Forceps/vacuum*	*6 (14.6%)*	*0*	*6 (12.0%)*	*20 (33.3%)*	*0*	*20 (19.6%)*

Only 5 (10%) of the 50 breech women had babies in a complete breech position; 4 of these had an OOH vaginal breech birth and the 5th transferred to a hospital for arrest at 7 cm. Of the 45 frank presentations, 36 gave birth vaginally OOH and the other 9 women (all primips) transferred in labor, leading to 2 operative vaginal breech births and 7 cesareans.

### In-labor transports

We do not have access to complete medical records for the in-labor transports, which limits our ability to analyze some outcomes on an intention-to-treat basis. All 10 in-labor breech transports were primiparas and were transported for arrested labor/descent at or beyond 6 cm (*n* = 7) or during second stage (*n* = 3) (Table 4). None were emergent when the decision to transport was made. Six had a non-emergent cesarean section upon admission; these babies did well and none required NICU admission. The seventh mother transferred for a stalled labor at 7 cm and prolonged rupture of membranes. She was afebrile with normal maternal vital signs and reassuring structured intermittent fetal auscultation at the time of recommended transfer. She transferred to a local hospital that did not offer the option of a vaginal breech birth and was thus admitted for a planned cesarean section with the fetal heartrate in the 150–160 s range without decelerations. The fetal monitoring was not felt to require urgent cesarean delivery; however, more than two hours after admission there was a prolonged bradycardia in the operating room and a cesarean was performed under spinal anesthesia. Neonatal resuscitation was unsuccessful.

**Table 4:** Indications for in-labor hospital transfer

Indication for transfer	Breech *n* = 50	Cephalic *n* = 102
Arrest of active labor (6–9 cm)	7	6
Arrest of labor in 2nd stage	3	
Maternal exhaustion and pain relief		3
Decelerations in early labor		2
Total in-labor transfers	10 (20.0%)	11 (10.8%)

The last three breech mothers were transported, all in stable condition, to a physician who offered the option of augmentation in one of the local hospitals. Two gave birth vaginally after epidural and oxytocin augmentation. Both of these births were prolonged, described as difficult, and entailed the use of Piper forceps and/or vacuum extraction and episiotomies with extensions. The third had an emergent cesarean section for fetal bradycardia immediately after placement of an intrauterine pressure catheter (IUPC). All 3 of these babies required NICU admission with 2 transported to another facility equipped with neonatal therapeutic hypothermia capability. Both babies born vaginally have recovered fully; the baby delivered by emergent CS has mild developmental delay.

There were 11 laboring cephalic women who were transported in labor, only one emergently. Six women transported for arrest of active labor. After epidural and oxytocin, two had spontaneous vaginal births, two had a vacuum extraction, and two had cesareans. One of the cesareans was due to a twin fetus papyraceous presenting in front of the live fetus and obstructing descent. (The mother had a selective fetal reduction early in pregnancy; her pregnancy was then treated as a singleton gestation.) The other cesarean occurred after a long delay at 10 cm with no descent. Three additional women transported for maternal exhaustion, one with back pain and asynclitism. All three had spontaneous vaginal births after epidural and oxytocin. The two final women were transported for audible decelerations in early labor. One was a multiparous woman with prolonged ROM and GBS+ status; she was transferred emergently and gave birth rapidly in the hospital. The other had a non-reassuring fetal heart rate tracing at 3 cm and was taken for cesarean section.

### Length of 2nd stage

We have partial data (27/50) on length of 2nd stage for the breech births that actually occurred OOH. In SJF’s practice, second stage is defined as beginning at complete dilation. Many breech and cephalic labors included a passive or “laboring down” phase before active maternal pushing began. Breech mothers were encouraged to labor down for as long as possible. SJF’s records do not distinguish between passive and active second stages. Of the 19 primiparous mothers, 2nd stage ranged from 19 to 228 min (mean: 94, SD: 58). Two of these 19 mothers had second stages exceeding 2 ½ hours (170 and 228 min). Of the 8 multiparous mothers, 2nd stage lasted between 5 and 34 min (mean: 17, SD 9.7). Second stage data are not available for the remaining breech mothers or for the cephalic births.

### Perineal integrity

We do not have full data on perineal outcomes for in-labor transports, so we can only report on completed OOH births (Tables 5 and 6). In the breech group there were 5 episiotomies performed plus two 3rd degree and one 4th degree laceration, all in primiparous mothers and repaired on site. None of the multiparous breech mothers required episiotomies or experienced any perineal trauma.

**Table 5:** Perineal outcomes of completed OOH births

Perineal integrity	Breech (*n* = 40)	Cephalic (*n* = 91)
Intact	25 (62.5%)	51 (56.0%)
1st degree tear	4 (10.0%)	23 (25.3%)
2nd degree tear	3 (7.5%)	5 (5.5%)
Episiotomy	5 (12.5%)	10 (11.0%)
3rd degree tear	2 (5.0%)	2 (2.2%)
4th degree tear	1 (2.5%)	0

**Table 6:** OOH breech perineal outcomes, categorized by parity and by maternal position

Perineal integrity	Parity		Maternal position	
	Primip (*n* = 31)	Multip (*n* = 9)	Upright (*n* = 11)	Lithotomy (*n* = 29)
Intact	16 (51.6%)	9 (100%)	10 (90.9%)	15 (51.7%)
1st degree tear	4 (12.9%)	0	1 (9.1%)	3 (10.3%)
2nd degree tear	3 (9.7%)	0	0	3 (10.3%)
Episiotomy	5 (16.1%)	0	0	5 (17.2%)
3rd degree tear	2 (6.5%)	0	0	2 (6.9%)
4th degree tear	1 (3.2%)	0	0	1 (3.4%)

Of the cephalic mothers giving birth OOH, a vacuum extractor was used 16 times and outlet forceps twice. Episiotomy was performed on 8 of these 18 operative vaginal deliveries. (This rate of operative delivery is skewed upward by calls to the author from local midwives requesting assistance for their own clients already in the second stage of labor.) There were two 3rd degree lacerations, both in the primiparous group and repaired on site, and no 4th degree lacerations.

### Estimated blood loss

We are missing data on estimated blood loss (EBL) for the majority of the breech and cephalic transports, thus we have calculated mean EBL for completed OOH births only. Mean EBL among the breech group was 314 ccs, compared to 386 ccs in the cephalic group; this difference was not statistically significant (*p* = 0.15). There were 4 breech mothers (10.0%) and 16 cephalic mothers (16.2%) with an EBL between 500 and 1000 ccs. No breech mother had an EBL > 1000 ccs and none required postpartum transport. Six cephalic women had an EBL > 1000 ccs; five of those stabilized at home and required no further acute treatment. The sixth mother was transported after birth by ambulance for an EBL of > 1500 ccs; once at the hospital, she stabilized with no need for blood transfusion and recovered without other morbidity.

### Apgar scores and neonatal morbidity

As with EBL, we only have information on Apgar scores for completed OOH births (Table 7). The rate of 1-min Apgars < 7 and mean 1- and 5-min Apgars were significantly different between the breech and cephalic groups. Although low 5-min Apgars were twice as common in the breech group, this difference was not significantly significant.

**Table 7:** 1- and 5- min Apgar scores for completed OOH births

OOH Apgar scores	Breech (*n* = 40)	Cephalic (*n* = 90^a^)	*P* value
1-min < 7	16 (40.0%)	7 (7.8%)	< 0.0001
5-min < 7	3 (7.5%)	3 (3.3%)	0.371
1-min average	6.3 (range 1–9)	8.0 (range 2–9)	< 0.0001
5-min average	8.4 (range 2–10)	8.9 (range 4–10)	0.0167

^a^ Apgar scores are missing for one baby in the cephalic group

All four Piper forceps assisted OOH breech births had 1-min scores < 7 but 5-min scores of 7 or better. Three other breech babies had 5-min scores < 7 with scores of 6, 6, and 2. This last baby had a terminal bradycardia for suspected umbilical cord compression just before birth and required respiratory assistance and cardiac resuscitation. Paramedics were called. By ten minutes the Apgar score was 8, the baby was doing well, and transport was deemed unnecessary.

Besides the three low 5-min Apgars, there were two neonatal morbidities in the breech group. One baby suffered a fractured humerus during an assisted all-fours breech birth. Pediatrics was consulted and no immediate attention was required. The infant recovered without incident. A second baby suffered a brachial plexus injury at an assisted breech delivery in lithotomy position for another terminal bradycardia just as the rump was protruding. She had momentary assisted ventilation. At the time of publication she is doing well but still has a significant residual Erb’s palsy 6 months after birth.

Seven cephalic babies had 1-min Apgar scores < 7 (7.8%) and 5 of these were vacuum assisted. There were only 3 cephalic babies with 5-min Apgar scores < 7, all vacuum assisted. The first woman had a prolonged second stage over 4 h, at which point the midwife requested SJF’s assistance. The second was a primiparous woman at 41.5 weeks with persistent occiput transverse position; assistance was requested by a local midwife and a vacuum vaginal birth was achieved over a midline episiotomy. The third baby was also a vacuum for a prolonged second stage and maternal exhaustion with a shoulder dystocia of < 1 min; the baby had a mild brachial plexus injury that has since resolved. All babies did well and none required transport.

### Postpartum transport

No breech mothers or babies required postpartum transport. In the cephalic group there was one newborn postpartum transport for persistent tachypnea; the infant had a spontaneous pneumothorax that resolved within 24 h. The other postpartum transport was the previously mentioned woman with an EBL of 1500 ccs that resolved without transfusion.

## Discussion

High rates of vaginal birth are possible for both breech and cephalic presentations in a home or birth center setting, similar to or greater than levels reported in two recent large studies of out-of-hospital births (Table 8; see also [42, 43]). The cesarean rate among our cephalic group was lower than reported in other home and birth data, but the rate of operative vaginal birth was much higher, likely due to SJF being called by local midwives to assist with obstructed 2nd stage labors, situations that otherwise would have required hospital transport.

**Table 8:** Outcomes compared to recent home and birth center studies (all numbers in %)

Source (*n* = pVBB at onset of labor)	Breech group (*n* = 50)	Cephalic group (*n* = 102)	Cheyney 2014 Home birth (*n* = 16,984)	Stapleton 2013 Birth center (*n* = 14,881)
In-labor TOC	20	10.8	10.9	12
PP TOC (mother or baby)	0	2	2.4	5
Cesarean section	16	2.9	5.2	6
Assisted vaginal birth	12	19.6	1.2	1
Spontaneous vaginal birth	72	77.5	93.6	93
5-min Apgar < 7	*7.5*	*3.3*	1.5	NR
EBL > 500 ccs	*10*	*16.2*	15.5	NR
EBL > 1000 ccs	*0*	*6.6*	4.8	NR
Intact perineum	*62.5*	*56*	49.2	NR
1st or 2nd degree	*17.5*	*30.8*	40.9	NR
Episiotomy	*12.5*	*11*	1.4	NR
3rd or 4th degree	*7.5*	*2.2*	1.2	NR

Italicized numbers indicate completed OOH births only (data not available for hospital transfers)

As expected, the cesarean rate was higher for the breech group than for the cephalic group. However, our breech group had lower cesarean rates compared to other home and hospital studies. The 222 planned home breech births reported in Cheyney 2014 had a vaginal birth rate of 57.2%, compared to 84% in this series. However, is unknown how many of the breech presentations in the Cheyney cohort were diagnosed before labor and thus how many were actually planned home breech births.

EBL was lower for the breech group and slightly higher for the cephalic group compared to the home births in Cheyney 2014. Women planning OOH generally have good perineal outcomes; 80% of the breech group, 86.8% of the cephalic group, and 90.1% of the Cheyney cohort experienced either moderate (intact perineum or 1st/2nd degree lacerations) or no perineal trauma. However, as stated previously, our data on perineal integrity include only the completed OOH births.

If we look at planned hospital VBB (Table 9), successful vaginal birth rates range from 56.7 to 71% in a sampling of single-center, multi-center, and national registry studies [1, 9, 15, 44–46]. We attribute the high vaginal success rates in our series to the collaboration between the obstetrician and midwife and to a setting that allowed the mother to have an undisturbed, physiological labor.

**Table 9:** Rate of successful vaginal birth and low Apgar scores compared to hospital studies of pVBB

Source	Country, dates	Type of study	# of pVBB	Vaginal birth rate	5-min Apgar < 7
Breech group	USA, 2010–2017	single center retro.	50	84.0%	7.5%^a^
TBT 2000	International, 1997–2000	multi center RCT	1042	56.7%	3.0%
PREMODA 2006	France & Belgium, 2001–2002	multi center pros.	2526	71.0%	1.48%
Vlemmix 2014	Netherlands, 1999–2007	registry retro.	27,817	58.7%	2.2%
Vistad 2015	Norway, 1991–2011	registry retro.	17,500	64.0%	2.4%
Burgos 2015	Spain, 2003–2012	single center retro.	891	57.5%	2.2%
Louwen 2017	Germany, 2004–2011	single center retro.	433	62.1%	2.5%

^a^ Completed OOH births only—data not available for hospital transfers

retro. = retrospective

pros. = prospective

With the exception of the PREMODA study, all studies listed in Table 9 had higher rates of low 5-min Apgars for pVBB compared to pCS. Our breech cohort followed a similar (albeit nonsignificant) pattern, although outcomes were compared to planned cephalic births rather than planned cesarean breech births.

Although our numbers are too small to calculate statistical significance, upright positioning for breech birth may be more protective to the perineum than on-the-back positioning. This mirrors the findings reported in Louwen 2017 [15] and warrants further examination.

There is disagreement in breech literature and guidelines on optimal length of second stage. The TBT allowed up to 3.5 h for 2nd stage [1]. In the PREMODA cohort, over 18% of women had a passive second stage longer than 1 h, while 94% of the active 2nd stages were under 30 min. However, oxytocin was used in the large majority of all births (8.9% of all labors were induced, and oxytocin was used in 74.1% of all non-induced labors); this high rate of oxytocin could have affected the duration of 2nd stage [9]. The SOGC guidelines, which draw heavily from the PREMODA study, recommend a total of 2 ½ hours with no more than 60 min of active 2nd stage [13]. In the Louwen study on upright vs supine breech, supine breeches averaged 1.77 h and upright breeches averaged 1.02 h for 2nd stage. 17.1% of the supine breeches and 7.4% of the upright breeches had a 2nd stage exceeding 3 h [15]. SJF follows a midwifery model of care for managing first and second stage, eschewing rigid time limits in favor of individualized care and ongoing assessment of maternal and fetal status. This approach explains some of the longer second stages with a small number of the breech primips. Decisions to transport (for breech) or to offer operative delivery (for cephalic) are based on the mother’s request—usually for exhaustion—or on SJF’s recommendations due to lack of fetal progress/descent.

Breech mothers seeking care with SJF were significantly more likely to be primips compared to cephalic mothers. Uotila et al. observed a similar difference in parity between their breech and cephalic cohorts and proposed it might be due to ECV having higher success rates in multips, thus leaving more primips with persistent breech presentations [47].

We found a significant difference in birth weights between the two groups. We hypothesize the difference is likely due to having an upper weight limit for breech babies but not for cephalic babies. However, Molkenboer found that term breech presentations had a significantly lower mean birth weight compared to matched cephalic presentations, suggesting a possible causal relationship between birth weight and type of presentation [48].

One difference in breech labor protocols between a hospital and OOH setting is in fetal monitoring techniques; at home, intermittent monitoring with a handheld Doppler is the standard of care. We encourage further research comparing intermittent with continuous fetal monitoring for breech labors.

The advisability of augmentation for breech labors is still debated. Although the TBT and the PREMODA study both allowed augmentation, neither the RCOG nor the SOGC guidelines recommend augmentation for uterine dystocia in a spontaneous labor without epidural analgesia [1, 8, 9, 13]. Our experience confirms that, at least for out-of-hospital breech labors, a cesarean section is more prudent than augmentation; all three transports admitted for augmentation in a hospital setting had complicated neonatal courses. Whatever the indication for hospital transfer, women deserve systemic collaboration, good communication, and a smooth, nonjudgmental transition from home or birth center to hospital. (See the transfer guidelines created by the Home Birth Summit Collaboration Task Force [49]).

## Strengths & Limitations

Although fetal and maternal outcomes for care transfers were usually reported back informally by the parents, medical records for these women were not available for review, making further analysis of these hospital transfers impossible. Thus we have incomplete data on the transfers and were unable to do an intention-to-treat analysis on some outcomes.

We recognize that our cephalic and breech groups are not directly comparable, not only due to presentation but also due to differences in parity and EFW restrictions. However, because of the inherent preferences of their client base, out-of-hospital practitioners do not generally have access to a large cohort of women with breech presentations who choose a planned cesarean. With these limitations in mind, we chose to set out-of-hospital breech outcomes in the context of cephalic births occurring during the same time period with the same obstetrician. Using a cephalic cohort as a comparison group also has precedent in the medical literature [47, 48, 50–57].

The relatively small sample size of both groups limits the ability to extrapolate our findings, and our numbers are underpowered for calculating the relatively rare events of severe morbidity or mortality. However, this is the largest study of planned OOH vaginal breech birth with a single care provider. It provides a rich glimpse into what is possible for a breech birth with a trained practitioner who follows clear protocol guidelines and who respects the physiological process of birth. On the other hand, SJF offers a unique service and most women seeking OOH birth will not have access to a highly skilled obstetrician.

The high rates of vaginal birth in both breech and cephalic groups in our series may not be achievable in most hospital settings. The midwifery model of care used in this series does not lend itself well to the high-volume, shift-oriented practices in many hospitals. Instead, the midwifery model stresses preventative care, well-developed relationships, longer prenatal visits, personalized attention, and uninterrupted one-on-one care during labor, birth, and postpartum.

The breech mothers in our study faced the added stress and disruption of their birth plans, being forced to change providers and/or planned location of birth last minute. The impact of this dramatic upheaval for the breech mothers was not independently assessed, but it should not be ignored as a factor potentially affecting outcomes.

We acknowledge that many consider breech birth to be high-risk and home or birth center birth to be an absolute contraindication for breech presentation. However, hospitals in many countries have consistently failed or refused to offer VBB since the TBT, despite evidence that it remains a reasonable option for well-selected women. Women continue to value vaginal birth highly. When hospitals or providers refuse to offer vaginal breech birth, some women will seek care outside the hospital.

We warn of the dangers of restricting or outlawing vaginal breech birth at home or birth centers; this will result in some women giving birth unassisted, which is arguably less safe than breech birth with a trained practitioner. Instead, we encourage a model where women are fully informed of the risks (both absolute and relative) and benefits of their options and are allowed to make the final decision of where and how to give birth. Two such models are Amish birth centers allowing breeches, twins, and vaginal birth after cesarean (VBAC) [58] and state midwifery regulations that do not force the midwife to abandon care for breech, twin, or VBAC clients.^1^

Informed consent is now a fundamental principle of modern medicine, law, and ethics. This includes access to the full range of information about a treatment’s risks, benefits, and alternatives and the patient’s ability to freely consent to or refuse a proposed treatment [59–65]. For women with breech presentations, this means the right to refuse surgery in favor of a vaginal breech birth. ACOG’s May 2016 practice bulletin strongly upholds pregnant women’s right to refuse medical treatment. It reads:

[A] decisionally capable pregnant woman’s decision to refuse recommended medical or surgical interventions should be respected. The use of coercion is not only ethically impermissible but also medically inadvisable because of the realities of prognostic uncertainty and the limitations of medical knowledge. As such, it is never acceptable for obstetrician–gynecologists to attempt to influence patients toward a clinical decision using coercion [32].

Forcing women to have cesareans for breech presentations also violates U.S. legal rulings that uphold the right of competent adults to refuse surgery.^2^

Breech birth requires training and skill; it deserves respect and caution but not fear. We strongly recommend that hospitals stop banning vaginal breech birth and that residency training programs reinstitute training in vaginal breech birth as a core obstetric skill. Even today, around 1/4 to 1/3 of all breech presentations are undiagnosed before labor [66], highlighting the need for skilled attendants when it might not be advisable or possible to do a cesarean.

## Conclusions

Planned vaginal breech birth should remain an accessible option for all women, especially taking into account the short-and long-term risks of cesarean section to the baby, the mother, and the mother’s future babies. While universal cesarean for breech might prevent a very small number of fetal deaths, it comes at the price of overriding maternal autonomy and subjecting both mother and baby to another set of risks—risks that she might not be comfortable with [67]. We cannot overemphasize the importance many women place on giving birth vaginally. Bassaw et al. eloquently conclude in their 2004 study of breech outcomes at a tertiary hospital in Trinidad:

A policy of planned vaginal birth for selected breech fetuses with a low threshold to proceed to caesarean section may still be in the best interest of both mother and child. The individual woman's wishes must be taken into consideration as for some, labour is an integral and treasured experience and a vaginal delivery is a life event of enormous magnitude [68].

As our study demonstrates, out-of-hospital vaginal breech birth with carefully selected patients, specific protocol guidelines, and a skilled provider results in high rates of vaginal birth and good maternal outcomes. However, the absolute risks of neonatal morbidity and mortality are difficult to quantify due to the small samples sizes in this study and to our inability to include some outcomes from hospital deliveries occurring after intrapartum transfer. Whether a planned OOH breech birth is considered reasonable or safe is an individualized judgment call based on the history and values of the expectant family and on the birth options available within their communities. Reviving vaginal breech skills in all settings and respecting maternal autonomy would benefit both practitioners and the women they care for.

## Abbreviations

CS: Cesarean section
EBL: Estimated blood loss
ECV: External cephalic version
EFW: Estimated fetal weight
IUFD: Intrauterine fetal demise
IUPC: Intrauterine pressure catheter
OOH: Out-of-hospital (i.e., home or birth center)
pCS: Planned cesarean section
pVBB: Planned vaginal breech birth
TBT: Term Breech Trial
TOC: Transfer of care
VBAC: Vaginal birth after cesarean
VBB: Vaginal breech birth

## Footnotes

1 See, for example, Wis. Admin. Code SPS § 182.03(4).

2 See, for example: Union Pacific Railway Co. v. Botsford, 141 U.S. 250, 251 (1891); Schloendorff v. Society of New York Hospital, 105 NE. 92, 93 (N.Y. 1914); Cruzan V. Director, Missouri Dept. of Health, 497 U.S. 261, 270 (1990); In re Brown, 478 So.2d 1033 (Miss. 1985); Cruzan V. Harmon, 160 S.W.2d 408, 417 (Mo. 1988); Matter of Guardianship of L.W., 482 N.W.2d 60, 65 (Wis. 1992); In re Fiori, 673 A.2d 905, 910 (Pa. 996); Stouffer v. Reid, 993 A.2d 104, 109 (Maryl. 2010).
